# AVPR2 is a potential prognostic biomarker and correlated with immune infiltration in head and neck squamous cell carcinoma

**DOI:** 10.1186/s12920-023-01500-3

**Published:** 2023-03-30

**Authors:** Linwei Mao, Zhiyong Pan, Wenzhi Chen, Weiqun Hu, Xiufen Chen, Huiting Dai

**Affiliations:** Department of Otolaryngology, Affiliated Hospital of Putian University, Putian, Fujian People’s Republic of China

**Keywords:** AVPR2, Prognostic biomarker, Immune, Head and neck squamous cell carcinoma

## Abstract

**Purpose:**

To explore the potential of AVPR2 in the immunotherapy of head and neck squamous cell carcinoma (HNSCC), thus providing insights into a novel antitumour strategy.

**Methods:**

In this study, we performed a comprehensive analysis of the AVPR2 gene in HNSCC using public datasets from The Cancer Genome Atlas and Gene Expression Omnibus. We explored the potential molecular mechanism of HNSCC in clinical prognosis and tumour immunity from the aspects of gene expression, prognosis, immune subtypes, and immune infiltration.

**Results:**

AVPR2 expression was significantly downregulated in primary HNSCC tissue compared with normal tissue. HNSCC patients with high AVPR2 expression had a better prognosis. Moreover, the results of GSEA showed that immune subtype surface AVPR2 is involved in immune modulation. Furthermore, significant strong correlations between AVPR2 expression and infiltrating immune cells existed in HNSCC, and marker genes of infiltrating immune cells were also significantly related to AVPR2 expression in HNSCC. These results suggest that AVPR2 expression can influence the infiltration of tumour immune cells. Finally, we found that only high levels of B-cell infiltration, rather than those of other immune cells, can predict a longer overall survival in patients with HNSCC. Future studies are needed to explore the role of AVPR2 and tumour-infiltrating B cells in HNSCC.

**Conclusions:**

The AVPR2 gene may be a prognostic biomarker of HNSCC. Moreover, AVPR2 may play a role in HNSCC immune modulation, and the regulation of tumour-infiltrating B cells by AVPR2 may be a key link.

## Introduction

According to the World Health Organization's most recent data, head and neck squamous cell carcinoma (HNSCC) is the eighth most common cancer globally. This disease is also the ninth leading cause of cancer-related mortality [1]. For patients with early HNSCC, either surgery or radiotherapy alone can achieve satisfactory results. However, the majority of patients in the locoregionally advanced stage when diagnosed need strategies to improve the efficacy of combination therapy. Despite the major advances in imaging techniques, surgery, radiotherapy, and chemotherapy in recent decades, the outcome in terms of survival remains unsatisfactory [2]. Therefore, further exploration of the molecular mechanism involved in the pathogenesis of HNSCC is essential for the development of innovative therapeutic approaches to improve survival.

Arginine vasopressin receptor 2 (AVPR2) belongs to the seven-transmembrane domain G protein-coupled receptor (GPCR) superfamily. This molecule is expressed primarily in the distal convoluted tubules and collecting ducts. Under physiological conditions, its main function is to concentrate urine and maintain water balance in the body by stimulating mechanisms in response to the pituitary hormone arginine pressor (AVP). Mutations in this gene are the most important cause of congenital nephrogenic diabetes insipidus [3]. The expression of AVPR2 has been reported in a variety of cancers, including osteosarcoma, renal cell carcinoma, breast cancer, pancreatic cancer, colorectal cancer, and small cell lung cancer [4–7]. Activation of AVPR2 promotes proliferation of clear cell renal carcinoma cell lines and is associated with tumour grade [8, 9]. However, in some other studies, AVPR2 may inhibit tumour proliferation by activating the canonical adenylate cyclase/cAMP/PKA axis in tumour cells [6, 10, 11].

In addition to its classical functions, AVPR2 may have immunomodulatory functions. Immune checkpoint inhibitors (ICISs) have made unprecedented progress in the treatment of cancer. Studies have shown that AVPR2 is significantly downregulated in patients with immune-related adverse events (Irae) after immunotherapy [12]. AVPR2 is associated with immune cell infiltration in renal cell carcinoma [5]. However, the role of AVPR2 in HNSCC is unclear.

In this study, we extensively investigated the prognostic and immunological role of AVPR2 in HNSCC. We also studied the potential link between AVPR2 expression and immune subtypes, promising immune biomarkers, and tumour-infiltrating immune cells in the tumour microenvironment. The purpose of this study was to explore the potential of AVPR2 in the immunotherapy of HNSCC, thus providing insights into a novel antitumour strategy.

## Methods

### Expression and gene alteration of AVPR2 in HNSCC

cBioportal (http://www.cbioportal.org/) is a powerful genomic analysis tool for the TCGA database [13]. The OncoPrint module was used to analyse the gene alteration and mRNA expression of AVPR2 in HNSCC. UALCAN (http://ualcan.path.uab.edu), a comprehensive web resource for analysing cancer omics data [14], was used to compare AVPR2 expression between HNSCC and normal tissues. In addition, the microarray data of the GSE59102 dataset, which was obtained from the Gene Expression Omnibus (GEO) database, were used for validation. GSE59102 included the whole human genome microarray analysis data of 29 tumour tissue samples and 13 marginal tissue samples of head and neck squamous cell carcinoma treated by surgical ablation. The t test was used to estimate the significance of differences in gene expression levels between groups, and *P* < 0.05 was considered statistically significant.

### Immunohistochemical staining

We collected clinical tissue specimens from 30 cases of squamous cell carcinoma of the head and neck (30 tumour tissues and 27 paracarcinoma tissues). The study was approved by the Ethics Committee of the Affiliated Hospital of Putian University. The expression of AVPR2 and CD40LG protein in these tissues was detected by immunohistochemistry. The primary antibodies used in the current studies were as follows: AVPR2 (YaJi Biological, Inc., Shanghai, China), and CD40LG (YaJi Biological, Inc., Shanghai, China). Tumour specimens were fixed in 4% paraformaldehyde, paraffin-embedded, cut into 4-μm sections, and then dewaxed. Antigen recovery was accomplished by pressure steaming at 95 °C for 3 min. Samples were incubated with diluted primary antibody at 37 °C for 1 h. The rest of the immunohistochemical procedures were performed according to the manufacturer's instructions, and the final results were estimated independently by two pathologists. For the semiquantitative assessment of AVPR2 staining, the immunoreactivity score (IRS) was used to investigate regional differences in staining. Ten typical high-power fields were randomly observed under light microscopy. The staining intensity and percentage of stained cells were evaluated for each sample, and the final IRS was calculated. Staining intensity (SI) was scored from 0 (no staining) to 3 (strong), and the percentage of positive cells (PP) was as follows: 0 when PP < 1%, 1 when PP = 1–10%, 2 when PP = 11–29%, 3 when PP = 30–60%, and 4 when PP ≥ 60%. The above two scores were multiplied together, and the IRS ranged from 0 to 12. For semiquantitative evaluation of CD40LG staining, the proportion of CD40LG cells was assessed by estimating the percentage of cells with strong intensity of membrane staining in the stroma cells. Statistical analysis and figure exhibition were performed using GraphPad Prism 7.0. Unpaired t tests were performed to measure the difference in continuous variables between groups. All tests were two-sided, and a *P* value < 0.05 was considered statistically significant**.**

### Analysis of the prognostic value of AVPR2 in HNSCC

The GEPIA database (http://gepia.cancer-pku.cn) and PrognoScan database (http://dna00.bio.kyutech.ac.jp/PrognoScan/index.html) were used to explore the prognostic value of AVPR2 expression in human cancers [15, 16]. The GEPIA database is an online website, and its analysed tumour and normal tissue data were from the TCGA database. We used the GEPIA database to explore the correlation between AVPR2 expression and overall survival (OS) and disease-free survival (DFS) in HNSCC. In the GEPIA database, the median AVPR2 expression was used as a cutoff value to classify groups, and hazard ratios (HRs) with corresponding 95% confidence intervals (CIs) and log-rank *P* values were calculated. Dataset GSE2837 on the PrognoScan website obtained from the GEO database was used to further verify the prognostic value of AVPR2. The prognostic value was considered statistically significant when the *P* value was < 0.05.

### Database applied to analyse AVPR2 expression in immune subtypes of HNSCC

The TISIDB database (http://cis.hku.hk/TISIDB) is an online integrated repository portal that collects human cancer data from the TCGA database [17]. The correlation of AVPR2 expression with immune subtypes of HNSCC was explored through the TISIDB database. When the *P* value was < 0.05, the difference was considered to be statistically significant.

### Analysis of the correlation Between AVPR2 expression and immune infiltrating cells and their marker genes

TIMER is a comprehensive resource for systematically analysing immune infiltration in different cancer types (https://cistrome.shinyapps.io/timer/) [18] that contains 10,897 samples across 32 cancer types from TCGA. Using gene modules, we analysed AVPR2 expression and the correlation of AVPR2 expression with the abundance of immune infiltrates, including B cells, CD4+ T cells, CD8+ T cells, neutrophils, macrophages, and dendritic cells. The impact of infiltration of 6 types of immune cells on the overall survival of HNSCC patients was also analysed using TIMER. The SCNA module was utilized to compare the tumour infiltration levels with different somatic copy number alterations (SCNAs) for AVPR2.

Correlations between AVPR2 expression and gene markers of tumour-infiltrating immune cells were explored via correlation modules in TIMER. The gene markers of tumour-infiltrating immune cells included markers of neutrophils, monocytes, TAMs, M1 macrophages, M2 macrophages, natural killer (NK) cells, dendritic cells (DCs), B cells, CD8+ T cells, T cells (general), T-helper 1 (Th1) cells, T-helper 2 (Th2) cells, follicular helper T (Tfh) cells, T-helper 17 (Th17) cells, Tregs, and exhausted T cells. The GEPIA2 database (http://gepia2.cancer-pku.cn/) was used to verify the above results. The correlation analysis module can analyse the immune cell signature gene lists of interest, and the normal tissue was compared. Differences with a *P* value < 0.05 were considered statistically significant.

### Analysis of the correlation between AVPR2 expression and immune-related genes

Consecutively, TIMER was used to explore the relationship between AVPR2 expression and immune-related genes. We comprehensively examined the relationship between several series of immune checkpoint molecules, including antigen presentation, cell surface receptors, ligands, cell adhesion, co-stimulators and co-inhibitors molecules, related to AVPR2. Differences with a *P* value < 0.05 were considered statistically significant.

### Database used to explore AVPR2 co-expression networks

LinkedOmics (http://www.linkedomics.org) is a publicly available portal that includes multiomics data from all 32 TCGA cancer types [19]. Pearson correlation was used to statistically analyse the co-expression of AVPR2 in HNSCC patients with HiSeq RNA sequencing from the TCGA database. The function module of LinkedOmics performs analysis of Gene Ontology and Panther/Reactome pathway enrichment by gene set enrichment analysis (GSEA). Gene Ontology (GO) was used to categorize the genes according to biological processes, cellular components, and molecular functions. The rank criterion was the false discovery rate, and 500 simulations were performed; enriched gene sets were postprocessed by both affinity propagation and weighted set cover methods to reduce redundancy.

We imported the top 50 significant genes that were positively correlated with AVPR2 expression and the top 50 significant genes that were negatively correlated into STRING (https://string-db.org/) and performed protein–protein interaction (PPI) analysis with the minimum required interaction score set to 0.4. The nodes that did not interact with other proteins were hidden, and TSV files were output. The files were imported into Cytoscape 3.9.1 to obtain core modules from the PPI network via the MCODE plugin to help us discover genes in the PPI network that are more closely associated with AVPR2.

## Results

### The mRNA expression levels and gene alterations of AVPR2 in HNSCC

**Fig. 1:**
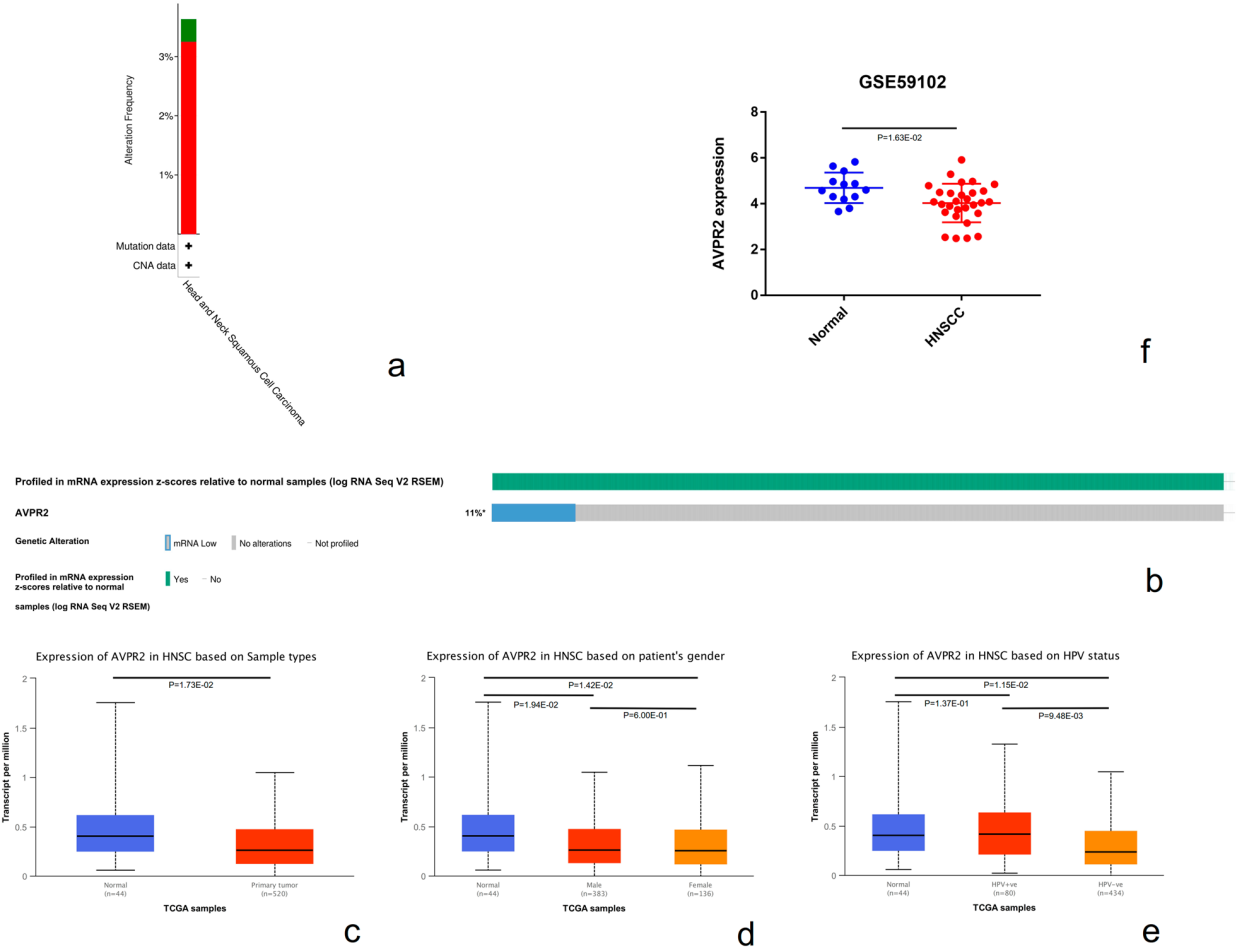
Expression and gene alteration of AVPR2 in HNSCC. **a** AVPR2 gene alteration types in HNSCC analysed by the cBioPortal database. **b** Oncoprint displays the mRNA expression levels of AVPR2 genes involved in HNSCC. **c** AVPR2 expression levels in normal and HNSCC tissues from the TCGA database analysed by UALCAN. Two factors, **d** sex and **e** HPV, were considered. **f** Differential expression data of AVPR2 between normal and HNSCC tissues from the GEO database

The cBioPortal website was used to investigate AVPR2 in HNSCC. The results showed that genomic alterations of AVPR2 occurred in 3.25% of patients with HNSCC. Among them, amplification was the most common type of mutation. (Fig. 1a). In addition, the overall oncoprint of AVPR2 gene expression in the HNSCC cohort (TCGA PanCancer) showed that mRNA expression was downregulated by 11.26% in all patients (Fig. 1b). Analysis of RNA-seq data from TCGA by UALCAN showed that AVPR2 expression was significantly downregulated in primary HNSCC tissue (n = 520) compared with normal tissue (n = 44) (*P* = 1.73E-02) (Fig. 1c). In addition, compared with that in the normal tissue (n = 44), the HPV (−) group (n = 434) expression was significantly decreased (*P* = 1.15E−02), while the HPV (+) group (n = 80) expression was not significantly different (*P* = 1.37E−01). There was also a significant difference between the HPV ( +) group and the HPV (-) group (*P* = 9.48E−03) (Fig. 1e). AVPR2 expression was lower in tissues from both males (*P* = 1.94E−02) and females (*P* = 1.42E−02) than in normal tissues; however, there was no significant difference between male and female patients (*P* = 6.00E−01) (Fig. 1d). GEO data further showed that AVPR2 expression was downregulated in HNSCC tissues (*P* = 1.63E−02) (Fig. 1f). Therefore, our data further support that AVPR2 was decreased in HNSCC, which might mainly originate from the alteration of DNA copy number.

**Fig. 2:**
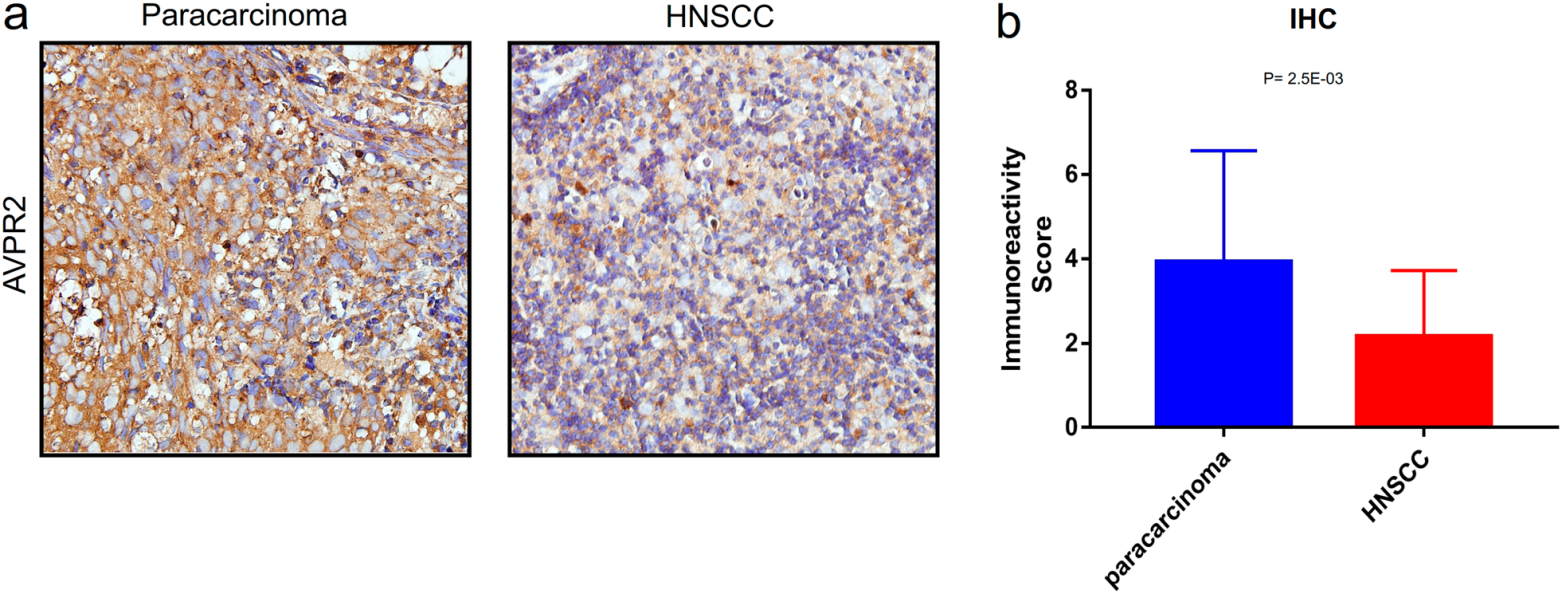
Validation of AVPR2 expression in HNSCC. **a** Representative microphotographs showing low and high AVPR2 staining intensity in HNSCC tissues and normal tissues. Bar = 100 μm. **b** Expression levels of AVPR2 in tumour and paracarcinoma tissues in HNSCC

To validate the AVPR2 expression results from public databases, we evaluated the expression of AVPR2 in head and neck squamous cell carcinoma tissues and paracarcinoma tissues by IHC (Fig. 2a). The results showed that the AVPR2 protein expression in HNSCC tissues was significantly lower than that in paracarcinoma tissues (*P* = 2.5E−03) (Fig. 2b).

### The role of AVPR2 in the survival of HNSCC patients

**Fig. 3:**
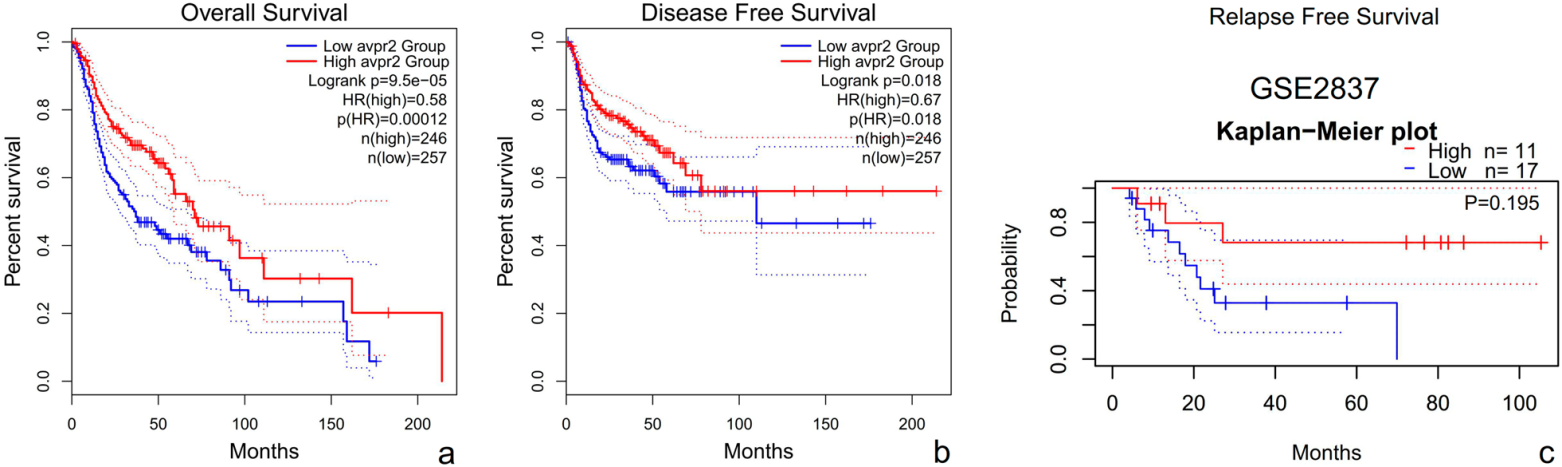
Kaplan‒Meier survival curves comparing high and low expression of AVPR2 in HNSCC. Kaplan‒Meier survival curve of **a** overall survival and **b** disease-free survival analysed by the GEPIA database. **c** Based on the GSE2837 dataset, an RFS rate analysis of HNSCC patients was performed

Subsequently, the online tool GEPIA was used to divide HNSCC samples into a high expression group and a low expression group according to the expression level of AVPR2 to study the correlation between AVPR2 and the prognosis of HNSCC patients. As shown in Fig. 3a, b, we observed that low AVPR2 expression was associated with poor prognosis in HNSCC patients. Overall survival (OS) was significantly poorer in patients with low AVPR2 expression than in those with high AVPR2 expression (*P* = 9.5E−05). A similar result was observed in the disease-free survival (DFS) analysis (*P* = 1.8E−02). Since GEPIA is a web server for analysing the RNA sequencing data from the TCGA database, to confirm the relationship between AVPR2 expression and HNSCC prognosis, we also used the GSE2837 dataset in the GEO database for verification. Although there was no significant difference due to the small sample size, the trend of relapse-free survival (RFS) in patients with high AVPR2 expression was still better than that of patients with low AVPR2 expression (Fig. 3c).

A Cox proportional hazard model was constructed to assess the prognostic value of AVPR2 expression in the overall survival of HNSCC patients using TIMER. After excluding the potential effects of age, sex, tumour stage, and tumour purity in 428 HNSCC patients, we found that compared with HNSCC patients with low AVPR2 expression, HNSCC patients with high AVPR2 expression had a lower risk of death (HR 0.484, 95% CI 0.293–0.798, *P* = 0.004) (Table 1).

**Table 1:** Cox proportional hazard model of HNSCC among 428 patients

Parameter	coef	HR	95%CI_lower	95%CI_upper	*P* value
Age	0.024	1.024	1.010	1.039	0.001
Gender (male)	− 0.140	0.870	0.627	1.206	0.402
Stage II	0.598	1.819	0.635	5.209	0.265
Stage III	0.753	2.122	0.742	6.074	0.161
Stage IV	1.225	3.405	1.254	9.246	0.016
Purity	− 0.188	0.829	0.409	1.678	0.601
AVPR2	− 0.726	0.484	0.293	0.798	0.004

coef, coefficient; CI, confidence interval; HR, hazard ratio

### AVPR2 expression is related to immune subtypes in HNSCC

**Fig. 4:**
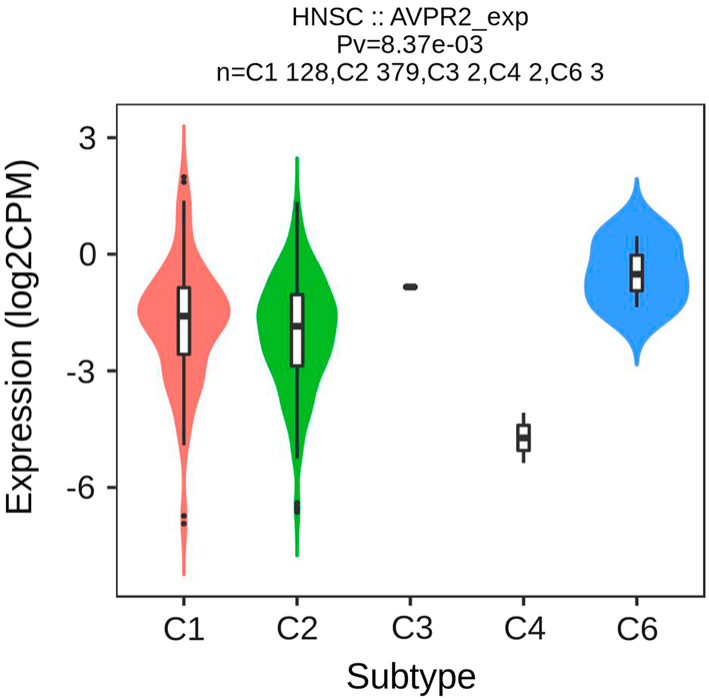
The relationship between AVPR2 expression and HNSCC immune subtypes

Subsequently, the Tisidb website was used to explore the role of AVPR2 expression in HNSCC immune subtypes. Immune subtypes were classified into 6 types: C1 (wound healing), C2 (IFN-gamma dominant), C3 (inflammatory), C4 (lymphocyte depleted), C5 (immunologically quiet), and C6 (TGF-b dominant) [20]. The results showed that there were 128 cases of C1, 379 cases of C2, 2 cases of C3, 2 cases of C4, 3 cases of C6 and no cases of C5, and AVPR2 expression was related to different immune subtypes in HNSCC (*P* = 8.37E-03) (Fig. 4).

### AVPR2 expression is associated with HNSCC immune infiltration and signature genes

**Fig. 5:**
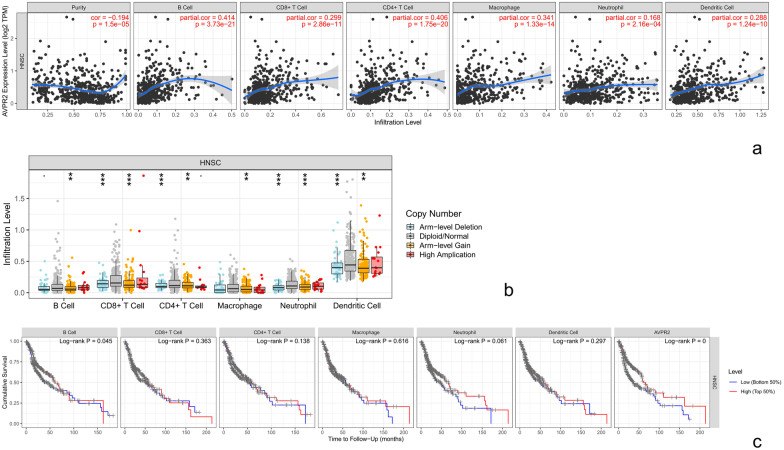
AVPR2 expression is associated with immune cell infiltration in HNSCC. **a** The correlation between AVPR2 expression and immune cell infiltration in HNSCC samples. **b** The correlation between somatic copy number alterations and immune cell infiltration. **c** Correlation between the infiltration of immune cells and HNSCC patient prognosis

Immune cells in the tumour microenvironment have an important impact on tumour progression. Therefore, using TIMER, we evaluated the relationship between AVPR2 expression and the immune infiltration level of HNSCC. The results showed that AVPR2 expression was negatively correlated with purity in HNSCC and positively correlated with the infiltration level of B cells, CD8+ T cells, CD4+ T cells, macrophages, neutrophils, and dendritic cells (Fig. 5a).

Subsequently, we analysed the correlation between different SCNAs of AVPR2 and immune cell infiltration in HNSCC samples. The immune infiltration level for each category was compared with the diploid/normal level. As shown in Fig. 5b, our data showed that SCNAs were significantly correlated with the infiltration of all 6 immune cells: B cells, CD8+ T cells, CD4+ T cells, macrophages, neutrophils, and dendritic cells. The level of immune cell infiltration appears to be highest in diploid/normal samples.

We also assessed the association between immune-infiltrating cells and outcomes in HNSCC patients. As shown in Fig. 5c, a high level of B-cell infiltration (*P* = 0.045) was significantly indicative of better clinical results, while macrophages, macrophages, neutrophils, and dendritic cells showed no significant differences.

Next, we used the TIMER database to study the correlation between the expression of AVPR2 and different signature gene subsets of immune cells in HNSCC. The immune cells in HNSCC were analysed, including T cells (general), CD8+ T cells, B cells, natural killer (NK) cells, dendritic cells, monocytes, tumour-associated macrophages (TAMs), M1 and M2 macrophages, and neutrophils. In addition, we analysed the T-cell subsets T helper cell 1 (Th1), T helper cell 2 (Th2), follicular helper T (TFH), T helper cell 17 (Th17), regulatory T (Treg), and exhausted T cells. Since the tumour purity of the sample impacts the immuno-infiltration analysis, the correlation analysis was adjusted accordingly. The analysis showed that the expression of AVPR2 in HNSCC was significantly correlated with the expression of signature genes in most (52/57) various immune cells and different T-cell subsets. Among the 35 signature genes of immune cells, 31 were significantly correlated with AVPR2 expression, and some of them showed moderate correlation, such as the B-cell markers CD19 (R = 0.484, *P* = 1.06E−27) and CD79A (R = 0.479, *P* = 2.27E−27), the neutrophil markers CCR7 (R = 0.502, *P* = 3.34E-30) and the dendritic cell markers CD1C (R = 0.403, *P* = 1.45E−18). Among the signature genes of 17T-cell subsets, 16 showed a correlation with the expression of AVPR2, among which STAT5A (R = 0.418, *P* = 6.06e−20) in Th2 cells showed a moderate correlation. In addition, the expression of AVPR2 showed a significant correlation with all 5 T-cell exhaustion types (Table 2).

**Table 2:** Correlation between AVPR2 and relate genes and markers of immune cells analyzed by TIMER

Description	Gene markers	None		Purity	
		Cor	*P* value	Cor	*P* value
Neutrophols	CCR7	0.523	***	0.502	***
	ITGAM	0.362	***	0.327	***
	CEACAM8	0.109	0.046	0.105	0.082
Natural killer cell	KIR3DL3	0.109	0.046	0.079	0.261
	KIR2DS4	0.085	0.138	0.054	0.495
	KIR3DL2	0.291	***	0.261	***
	KIR3DL1	0.213	***	0.204	***
	KIR2DL4	0.171	**	0.149	*
	KIR2DL3	0.176	**	0.144	0.011
	KIR2DL1	0.143	*	0.124	0.038
Monocyte	CSF1R	0.390	***	0.345	***
	CD86	0.220	***	0.165	**
M1 Macrophage	NOS2	0.322	***	0.344	***
	PTGS2	− 0.097	0.057	− 0.062	0.333
	IRF5	0.237	***	0.224	***
M2 Macrophage	CD163	0.292	***	0.253	***
	MS4A4A	0.284	***	0.232	***
	VSIG4	0.269	***	0.223	***
TAM	CCL2	0.424	***	0.379	***
	IL10	0.326	***	0.284	***
	CD68	0.107	0.034	0.061	0.338
Dendritic cell	CD1C	0.450	***	0.403	***
	HLA-DPB1	0.406	***	0.361	***
	HLA-DPA1	0.362	***	0.314	***
	HLA-DRA	0.347	***	0.297	***
	ITGAX	0.328	***	0.289	***
	HLA-DQB1	0.308	***	0.275	***
	NRP1	0.220	***	0.178	**
B cell	CD19	0.504	***	0.484	***
	CD79A	0.495	***	0.479	***
T cell (general)	CD3E	0.429	***	0.390	***
	CD2	0.424	***	0.387	***
	CD3D	0.396	***	0.353	***
CD8+ T cell	CD8B	0.365	***	0.331	***
	CD8A	0.314	***	0.271	***
Th1	TBX21	0.358	***	0.322	***
	STAT4	0.254	***	0.213	***
	STAT1	0.061	0.243	0.017	0.828
	IFNG	0.182	**	0.135	*
	TNF	0.144	*	0.128	0.014
Th2	STAT5A	0.437	***	0.418	***
	GATA3	0.273	***	0.245	***
	IL13	0.237	***	0.210	***
	STAT6	0.164	**	0.180	**
Th17	STAT3	0.257	***	0.240	***
	IL17A	0.245	***	0.220	***
Treg	FOXP3	0.396	***	0.361	***
	STAT5B	0.336	***	0.326	***
	CCR8	0.338	***	0.304	***
	TGFB1	− 0.103	0.043	− 0.119	0.026
Tfh	BCL6	0.278	***	0.325	***
	IL21	0.296	***	0.260	***
T cell exhaustion	PDCD1	0.350	***	0.310	***
	CTLA4	0.329	***	0.295	***
	HAVCR2	0.286	***	0.239	***
	LAG3	0.232	***	0.200	***
	GZMB	0.212	***	0.170	**

TAM, tumor-associated macrophage; Th, T helper cell; Tfh, Follicular helper T cell; Treg, regulatory T cell; Cor, R value of Spearman’s correlation; None, correlation without adjustment. Purity, correlation adjusted by purity

*P < 0.01; **P < 0.001; ***P < 0.0001

We verified the above relationship with the GEPIA2 database, and the results were essentially the same for both databases. The results of GEPIA2 showed that AVPR2 expression was correlated with the expression of all immune cell and T-cell subset signature gene lists except M1 macrophages. In normal tissues, only the M2 macrophage and TAM signature gene list showed a significant correlation. These results strongly suggest that AVPR2 expression is associated with immune cell infiltration in the HNSCC tumour microenvironment (Table 3).

**Table 3:** Correlation between AVPR2 and the list of signature genes of all immune cells analyzed by GEPIA2

Description	Signature genes list	Tumor		Normal	
		Cor	*P* value	Cor	*P* value
Neutrophols	CCR7 ITGAM CEACAM8	0.32	***	0.23	0.13
Natural killer cell	KIR3DL3 KIR2DS4 KIR3DL2 KIR3DL1 KIR2DL4 KIR2DL3 KIR2DL1	0.096	0.029	− 0.12	0.44
Monocyte	CSF1R CD86	0.21	***	0.14	0.36
M1 Macrophage	NOS2 PTGS2 IRF5	0.07	0.11	− 0.11	0.48
M2 Macrophage	CD163 MS4A4A VSIG4	0.2	***	0.52	**
TAM	CCL2 IL10 CD68	0.24	***	0.33	0.027
Dendritic cell	CD1C HLA-DPB1 HLA-DPA1 HLA-DRA ITGAX HLA-DQB1 NRP1	0.26	***	0.12	0.44
B cell	CD19 CD79A	0.3	***	− 0.12	0.42
T cell (general)	CD3E CD2 CD3D	0.26	***	− 0.24	0.12
CD8+ T cell	CD8B CD8A	0.2	***	− 0.21	0.17
Th1	TBX21 STAT4 STAT1 IFNG TNF	0.15	**	− 0.099	0.52
Th2	Th2 Th2 Th2 Th2	0.23	***	0.055	0.72
Th17	STAT3 IL17A	0.15	**	− 0.18	0.23
Treg	FOXP3 STAT5B CCR8 TGFB1	0.22	***	0.051	0.74
Tfh	BCL6 IL21	0.19	***	0.11	0.47
T cell exhaustion	PDCD1 CTLA4 HAVCR2 LAG3 GZMB	0.16	**	− 0.097	0.53

Tumor, correlation analysis in tumor tissue of TCGA. Normal, correlation analysis in normal tissue of TCGA

*P < 0.01; **P < 0.001; ***P < 0.0001

### AVPR2 expression is associated with HNSCC immune-related genes

In view of the crucial role of immune checkpoint molecules in the regulation of tumour immunity [21], we thoroughly examined the correlation between these immune-related genes and AVPR2. The expression of AVPR2 in HNSCC was shown to be generally correlated with the levels of several series of immune checkpoint molecules related to antigen presentation, cell surface receptors, ligands, cell adhesion, co-stimulators and co-inhibitor molecules (Table 4).

**Table 4:** Correlation between AVPR2 and immune-related genes analyzed by TIMER

Gene Symbols	Function	Immune Checkpoint	None		Purity	
			Cor	*P* value	Cor	*P* value
HLA-DPB1	Antigen presentation	N/A	0.406	***	0.361	***
HLA-DPA1	Antigen presentation	N/A	0.362	***	0.314	***
HLA-DQA1	Antigen presentation	N/A	0.350	***	0.312	***
HLA-DRA	Antigen presentation	N/A	0.347	***	0.297	***
HLA-DQB2	Antigen presentation	N/A	0.319	***	0.285	***
HLA-DRB1	Antigen presentation	N/A	0.327	***	0.281	***
HLA-DRB5	Antigen presentation	N/A	0.314	***	0.276	***
HLA-DQB1	Antigen presentation	N/A	0.308	***	0.275	***
HLA-DQA2	Antigen presentation	N/A	0.317	***	0.270	***
MICB	Antigen presentation	N/A	0.099	0.024	0.091	0.043
HLA-B	Antigen presentation	N/A	0.085	0.052	0.045	0.321
HLA-A	Antigen presentation	N/A	0.068	0.120	0.043	0.344
MICA	Antigen presentation	N/A	0.016	0.710	0.040	0.375
HLA-C	Antigen presentation	N/A	0.074	0.092	0.032	0.479
SELP	Cell adhesion	Stimulatory	0.715	***	0.700	***
ITGB2	Cell adhesion	Stimulatory	0.427	***	0.382	***
ICAM1	Cell adhesion	Stimulatory	0.199	***	0.164	**
VTCN1	Co-inhibitor	Inhibitory	0.264	***	0.296	***
SLAMF7	Co-inhibitor	Inhibitory	0.301	***	0.245	***
BTN3A1	Co-inhibitor	Stimulatory	0.263	***	0.230	***
BTN3A2	Co-inhibitor	Stimulatory	0.262	***	0.224	***
C10orf54	Co-inhibitor	Inhibitory	0.247	***	0.202	***
CD276	Co-inhibitor	Inhibitory	− 0.040	0.365	− 0.042	0.357
PDCD1LG2	Co-inhibitor	N/A	0.086	0.050	0.029	0.517
CD274	Co-inhibitor	Inhibitory	0.043	0.322	− 0.004	0.922
CD28	Co-stimulator	Stimulatory	0.455	***	0.420	***
ICOSLG	Co-stimulator	Stimulatory	0.324	***	0.318	***
CD80	Co-stimulator	Stimulatory	0.156	**	0.111	0.014
CD40LG	Ligand	Stimulatory	0.493	***	0.461	***
IL1A	Ligand	Stimulatory	− 0.319	***	− 0.353	***
TNFSF4	Ligand	Stimulatory	0.326	***	0.315	***
CX3CL1	Ligand	Stimulatory	0.309	***	0.309	***
IL10	Ligand	Inhibitory	0.326	***	0.284	***
IL12A	Ligand	Stimulatory	0.240	***	0.246	***
IL2	Ligand	Stimulatory	0.280	***	0.242	***
IL13	Ligand	Inhibitory	0.237	***	0.210	***
IFNA1	Ligand	Stimulatory	− 0.155	***	− 0.171	**
VEGFB	Ligand	Inhibitory	0.138	*	0.168	**
IL1B	Ligand	Stimulatory	− 0.135	*	− 0.157	**
cxcL9	Ligand	Stimulatory	0.198	***	0.147	*
VEGFA	Ligand	Inhibitory	− 0.187	***	− 0.146	*
CCL5	Ligand	Stimulatory	0.191	***	0.143	*
IFNG	Ligand	Stimulatory	0.182	***	0.135	*
TNF	Ligand	Stimulatory	0.144	*	0.128	*
IL4	Ligand	Inhibitory	0.125	*	0.126	*
TGFB1	Ligand	Inhibitory	− 0.103	0.018	− 0.119	*
CD70	Ligand	Stimulatory	0.082	0.060	0.061	0.175
TNFSF9	Ligand	Stimulatory	− 0.048	0.277	− 0.052	0.253
CXCL10	Ligand	Stimulatory	0.063	0.149	0.018	0.696
IFNA2	Ligand	Stimulatory	0.001	0.990	0.014	0.758
ENTPD1	Other	Stimulatory	0.515	***	0.495	***
PRF1	Other	Stimulatory	0.266	***	0.221	***
GZMA	Other	Stimulatory	0.241	***	0.193	***
IDO1	Other	Inhibitory	0.182	***	0.155	**
HMGB1	Other	Stimulatory	0.043	0.331	0.062	0.167
ARG1	Other	Inhibitory	0.004	0.927	− 0.018	0.687
ADORA2A	Receptor	Inhibitory	0.549	***	0.524	***
EDNRB	Receptor	Inhibitory	0.505	***	0.501	***
BTLA	Receptor	Inhibitory	0.512	***	0.490	***
CD27	Receptor	Stimulatory	0.505	***	0.481	***
TNFRSF4	Receptor	Stimulatory	0.444	***	0.437	***
TLR4	Receptor	Stimulatory	0.450	***	0.410	***
TNFRSF14	Receptor	Stimulatory	0.418	***	0.391	***
CD4	Receptor	Stimulatory	0.417	***	0.374	***
TIGIT	Receptor	Inhibitory	0.380	***	0.342	***
PDCD1	Receptor	Inhibitory	0.350	***	0.310	***
CTLA4	Receptor	Inhibitory	0.329	***	0.295	***
TNFRSF18	Receptor	Stimulatory	0.256	***	0.292	***
IL2RA	Receptor	Stimulatory	0.326	***	0.288	***
Icos	Receptor	Stimulatory	0.307	***	0.264	***
TNFRSF9	Receptor	Stimulatory	0.299	***	0.263	***
HAVCR2	Receptor	Inhibitory	0.286	***	0.239	***
LAG3	Receptor	Inhibitory	0.232	***	0.200	***
KIR2DL3	Receptor	Inhibitory	0.176	***	0.144	*
KIR2DL1	Receptor	Inhibitory	0.143	*	0.124	*

Cor, R value of Spearman’s correlation; None, correlation without adjustment. Purity, correlation adjusted by purity

*P < 0.01; **P < 0.001; ***P < 0.0001

**Fig. 6:**
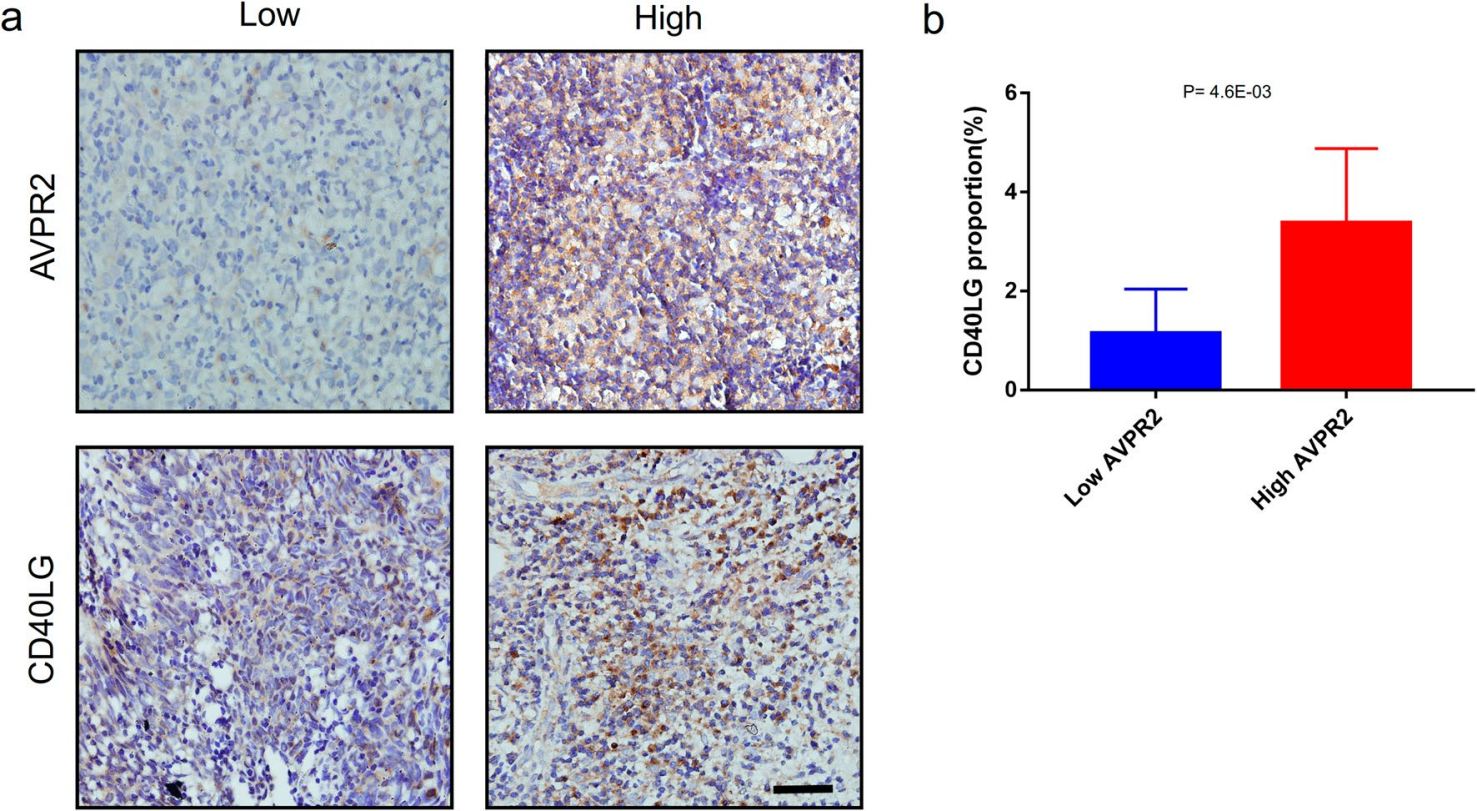
Validation of the immunological role of AVPR2 in HNSCC. **a** Representative microphotographs representing low and high AVPR2 and CD40LG staining intensities in HNSCC tissues and paracarcinoma tissues. Bar = 100 μm. **b** Expression of CD40LG in the low and high AVPR2 groups in HNSCC

CD40LG is an important co-stimulatory molecule that plays a key role in B-cell activation [22], and high CD40LG expression in HNSCC predicts a better prognosis [23]. Since we found a moderate correlation between CD40LG mRNA expression and AVPR2 in the public database (Cor = 0.461, *P* < 0.0001), we examined the expression level of CD40LG protein in HNSCC tissues using IHC staining for further validation (Fig. 6a). Next, the current HNSCC cohort was divided into low expression (IRS < 3) and high expression (IRS ≥ 3) groups based on the approximate median level of AVPR2 expression. Notably, the expression of CD40LG was higher in the high AVPR2 group than in the low AVPR2 group (*P* = 2.5E-03) (Fig. 6b). This result further suggests an immunological role for AVPR2 in HNSCC, suggesting that targeting AVPR2 may improve immunotherapy of HNSCC.

### The co-expression network of AVPR2 is associated with the immune response

**Fig. 7:**
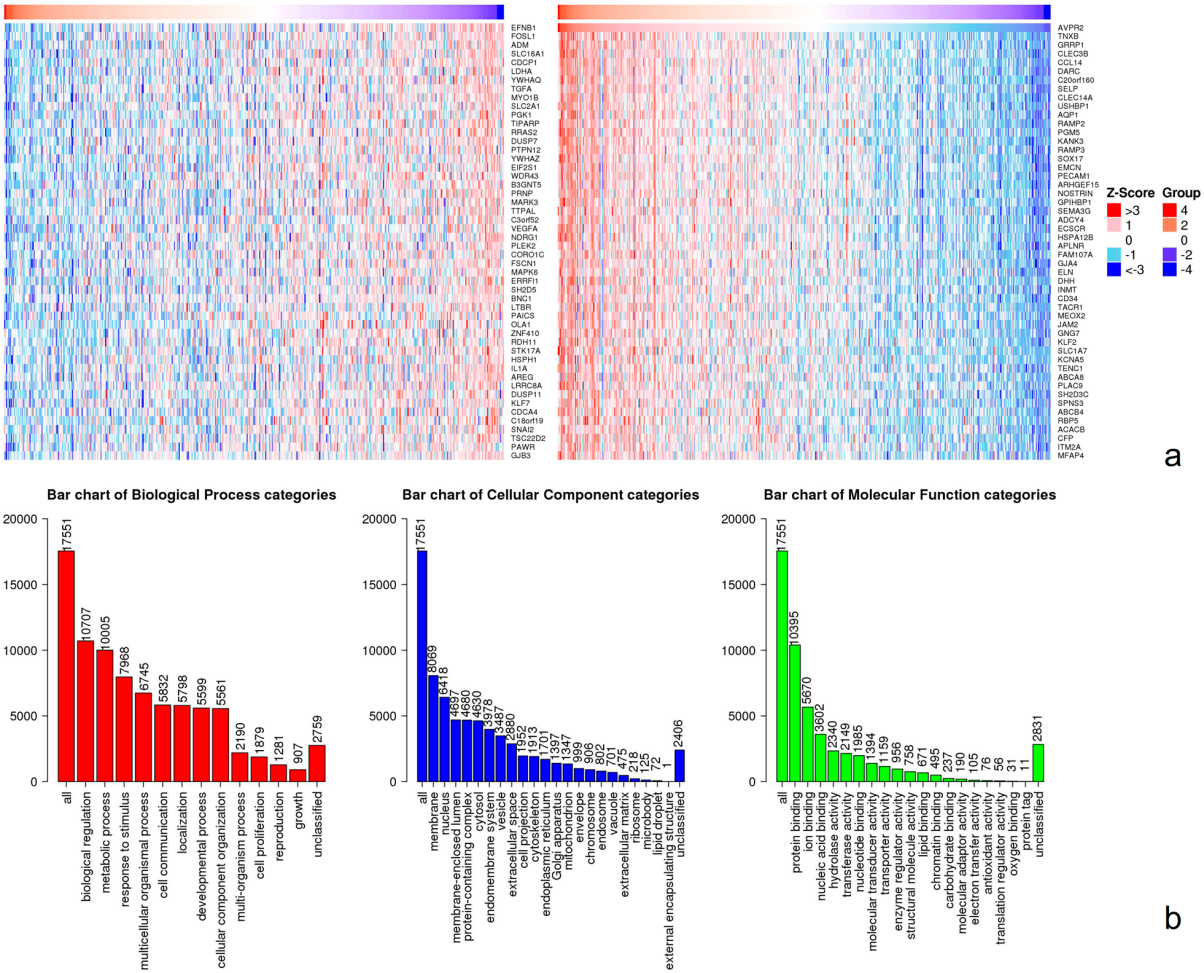
AVPR2 co-expression genes in HNSCC analysed by the LinkedOmics database. **a** Heatmaps showing the top 50 genes positively and negatively correlated with AVPR2 in HNSCC. **b** The Gene Ontology slim summary

To further explore the molecular mechanism of the AVPR2 gene in tumorigenesis, we attempted to identify its co-expression with AVPR2 by using the LinkedOmics method. Of the 20,032 genes, 2,891 were significantly positively correlated with CAPG, while 3,691 were significantly negatively correlated. The heatmap in Fig. 7a shows the top 50 significant genes with a positive correlation with AVPR2 expression (such as TNXA, GRRP1, CLEC3B, etc.) and 50 significant genes with a negative correlation (such as EGNB1, FOSL1, and ADM). The Gene Ontology (GO) slim summary is based upon the 17,551 unique Entrez Gene IDs; each biological process, cellular component, and molecular function category is represented by a red, blue, and green bar, respectively (Fig. 7b).

**Fig. 8:**
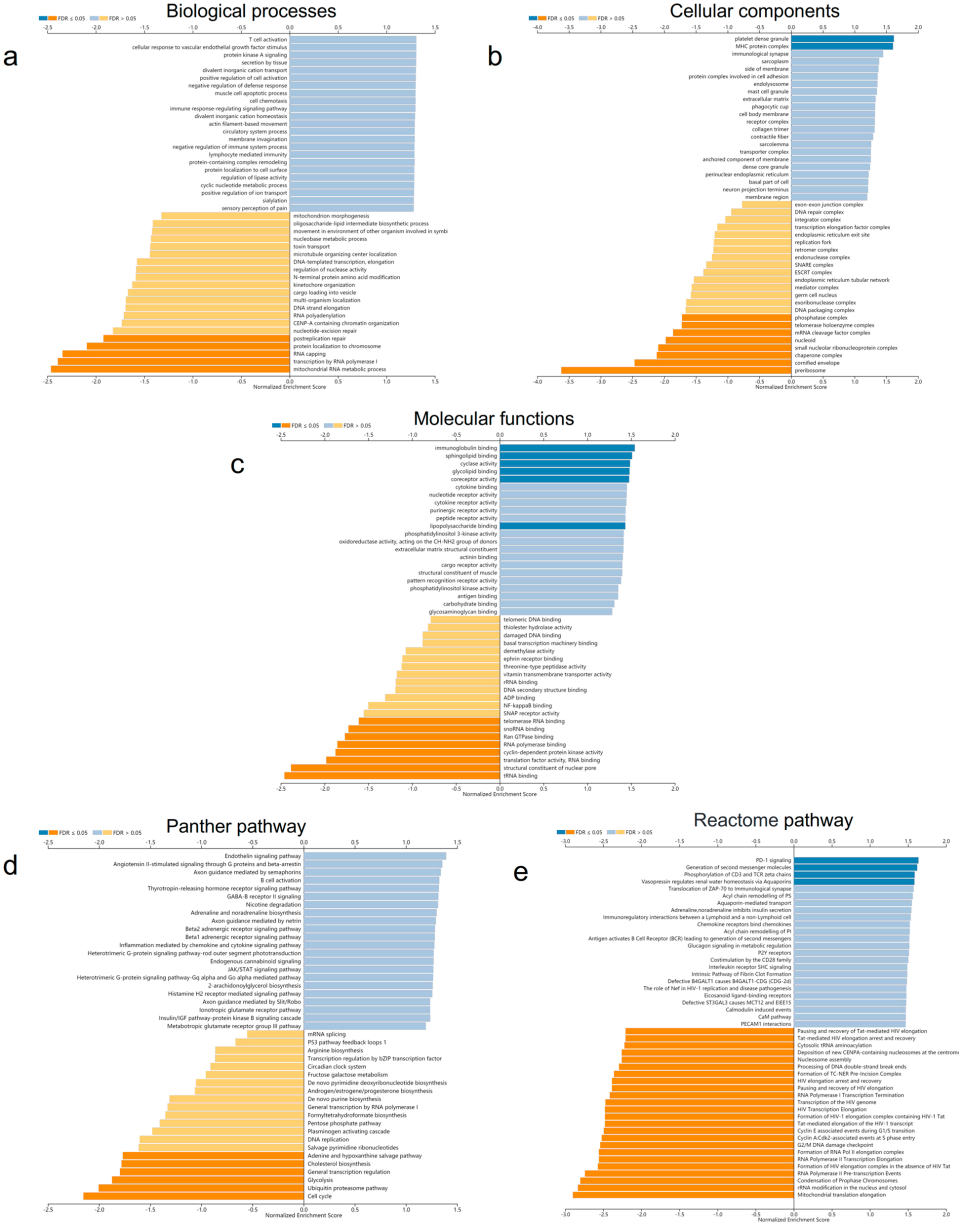
Functional enrichment analysis of AVPR2, including **a** GO biological process, **b** GO cellular components, **c** GO molecular function, **d** Panther pathways, and **e** Reactome pathways

Subsequently, we further performed gene set enrichment analysis (GSEA) on the AVPR2 co-expression dataset to determine the differentially activated Gene Ontology and signalling pathways in HNSCC. As shown in Fig. 8, the top 5 significantly enriched biological processes were T-cell activation, cellular response to vascular endothelial growth factor stimulus, protein kinase A signalling, secretion by tissue, actin filament-based movement, and divalent inorganic cation transport. The top 5 significantly enriched cellular components were platelet dense granule, MHC protein complex, immunological synapse, sarcoplasm, and side of membrane. The top 5 significantly enriched molecular functions were immunoglobulin binding, sphingolipid binding, cyclase activity, glycolipid binding, and coreceptor activity.

Furthermore, Panther pathway analysis showed the top 5 significantly enriched pathways: the endothelin signalling pathway, angiotensin II-stimulated signalling through G proteins and beta-arrestin, axon guidance mediated by semaphorins, B-cell activation, and the thyrotropin-releasing hormone receptor signalling pathway. Reactome pathway analysis showed significantly enriched pathways: PD-1 signalling, generation of second messenger molecules, generation of second messenger molecules, vasopressin regulation of renal water homeostasis via aquaporins, and translocation of ZAP-70 to immunological synapses. The above results show that AVPR2 plays an important role in the immune regulation and cell metabolism of HNSCC.

### Interaction between co-expressed genes and AVPR2

**Fig. 9:**
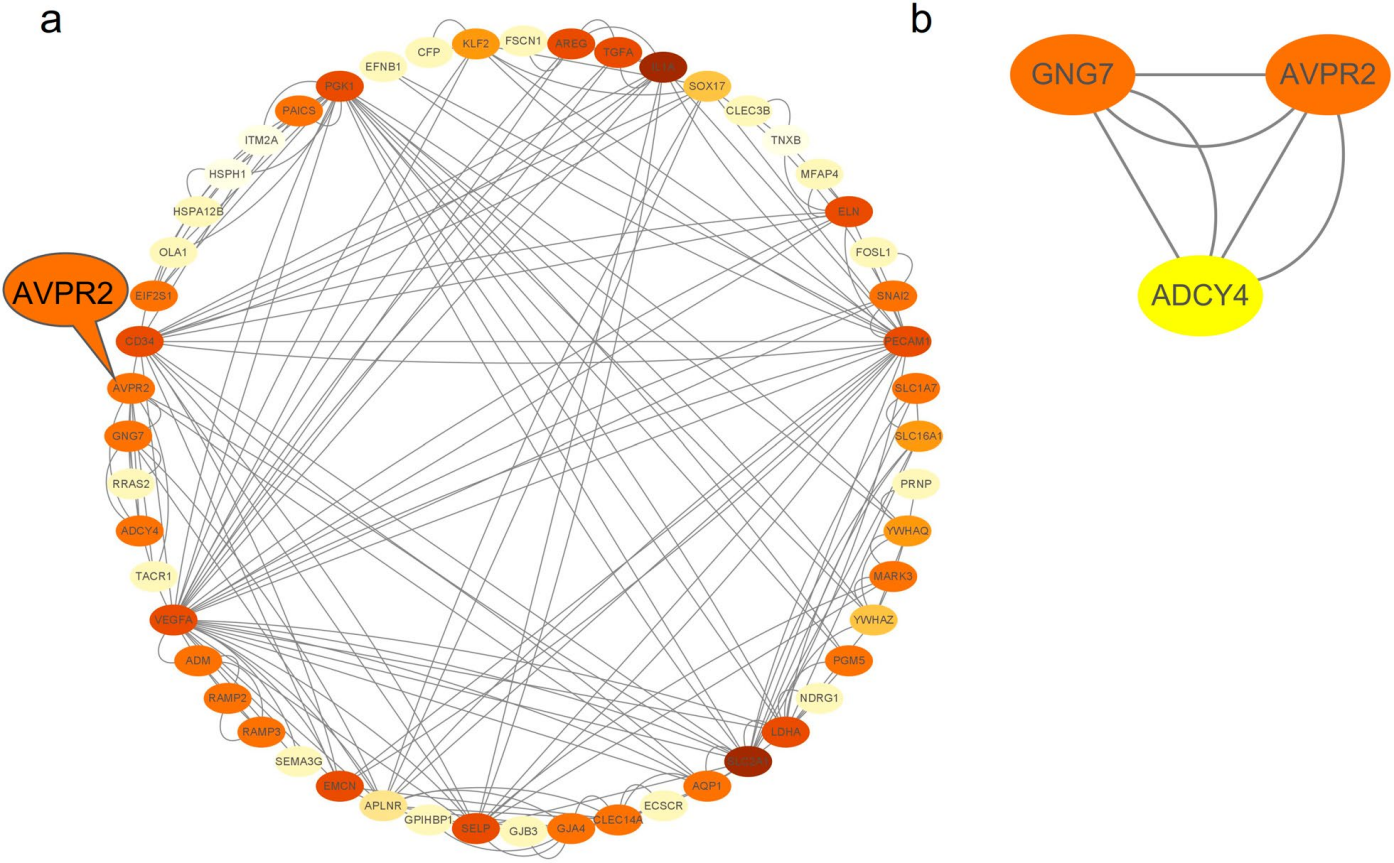
PPI network of AVPR2-related genes in HNSCC. **a** PPI network based on co-expressed genes. **b** PPI network based on co-expressed genes via the MCODE plugin

We constructed a PPI network using the STRING database and obtained core modules from the PPI network via the MCODE plugin to better understand the interactions between these co-expressed genes and AVPR2 (Fig. 9a). GNG7 and ADCY4 were more closely linked to AVPR2 and had a closer interrelationship (Fig. 9b). This finding suggests that they may be potential upstream or downstream genes of AVPR2, and the specific mechanism involved in their interaction with AVPR2 should be further investigated.

## Discussion

The expression of AVPR2 has been reported in a variety of cancers. In some studies, AVPR2 can promote tumour cell proliferation [8], but in other studies, it plays a protective role [5, 6, 11]. In addition, recent reports have shown that the AVPR2 gene may play an important role in tumour immunity [5, 12, 24]. To date, the role of AVPR2 in the occurrence and development of HNSCC has not been reported. Therefore, we comprehensively analysed the AVPR2 gene in HNSCC based on information extracted from various databases, including data related to gene expression, prognostic value, and the immune microenvironment.

The expression level of AVPR2 in renal cell carcinoma was significantly lower than that in normal tissues, and a high expression level of AVPR2 was associated with a better prognosis [5]. Furthermore, in AVPR2-expressing tumours, agonists that selectively act on AVPR2 exhibit robust antitumour activity [6, 11]. In this study, we used a variety of online tools to analyse HNSCC data based on high-throughput RNA sequencing data from the TCGA database and found that AVPR2 was significantly downregulated in HNSCC tissues compared with normal tissues and verified by immunohistochemistry. Moreover, survival analysis showed that AVPR2 expression was related to the prognosis of HNSCC patients. Low AVPR2 expression predicted poor prognosis in HNSCC patients, which was consistent with previous studies. Cox regression analysis further showed that AVPR2 was an independent prognostic factor of HNSCC. These results strongly suggest that AVPR2 is a potential prognostic marker for HNSCC.

Liao et al. showed that in addition to its classical functions, AVPR2 exhibits encouraging immunomodulatory functions in renal cell carcinoma [5]. AVP receptors (AVPR1a, AVPR1b and AVPR2) activated by AVP are involved in regulating CD4+ T-cell differentiation and mediating immune responses in target organs [24]. To determine the role of AVPR2 in HNSCC, we performed GSEA of AVPR2 co-expressed genes in HNSCC and revealed that genes related to AVPR2 expression were significantly enriched in multiple immune-related pathways, including the T-cell receptor signalling pathway, B-cell receptor signalling pathway, antigen processing and presentation, immune checkpoint, and other immune-related pathways. Then, to further elucidate the role of AVPR2 in tumour immunity, we explored the relationship between AVPR2 expression and different immune subtypes of HNSCC. The analysis shows that most of the immune subtypes in HNSCC are concentrated in C1 (wound healing) and C2 (IFN-γ dominant), which share several common characteristics, including a low Th1/Th2 cell ratio, high proliferation rate, and high intratumoral heterogeneity [20]. The expression of AVPR2 is significantly correlated with HNSCC immune subtypes, suggesting that AVPR2 may play a role in HNSCC immune modulation.

The tumour microenvironment is the "soil" for tumour survival, which has a strong influence on tumour growth and metastasis. Tumour-infiltrating immune cells have been shown to be an independent predictor of prognosis and immunotherapy efficacy in HNSCC patients [25]. We found that AVPR2 was positively correlated with all immune cells, including B cells, CD8+ T cells, CD4+ T cells, macrophages, neutrophils, and dendritic cells, using the TIMER database. By analysing the relationship between SCNAs and immune cell infiltration, we found that the immune infiltration level of samples with somatic copy number alterations seemed to be lower, suggesting that alterations in somatic copy number may be involved in the regulation of tumour immune cell infiltration. We also observed that AVPR2 was associated with the majority of tumour-infiltrating immune cell markers and immune-related genes， and that the expression of CD40LG, which plays a key role in B-cell activation, was positively correlated with AVPR2. Finally, we determined that only high infiltration of B-cells, rather than other immune cells, can predict a longer overall survival in patients with HNSCC. Therefore, it is important to further explore the role of AVPR2 and tumour-infiltrating B cells in HNSCC. Multidimensional interactions between B cells and other immune cells exist in the immune microenvironment [26]. Studies have indicated that increased levels of CD8+ and CD4+ TILs colocalizing with B-cell infiltration are related to long-term survival in NSCLC [27, 28]. The colocalization of CD8+ T cells and CD20+ B cells in melanoma predicts higher patient survival, and tertiary lymphoid structures play a key role in the immune microenvironment of melanoma [29]. In addition, the study found that there are tertiary lymphatic structures in HNSCC, which have definite T-cell zones, B-cell-rich follicles, and dendritic cells. Follicular DCs promote the development, class switching, and maturation of naive B cells in TLSs. This spatial organization of immune cells and tumour-infiltrating B cells are both related to the better prognosis of HNSCC [30, 31]. These results possibly explain the protective role of AVPR2 in HNSCC. Hence, our results suggest that AVPR2 may influence HNSCC tumour immunity primarily by modulating the tumour immune microenvironment, with AVPR2 regulation of tumour-infiltrating B cells possibly being a key link.

Although we conducted a systematic analysis of AVPR2 and cross-validated it using multiple databases, this study had some limitations. First, there are differences in microarray and sequencing data from different databases, which may lead to systemic bias. Second, based on bioinformatics methods and immunohistochemical assays, we concluded that AVPR2 expression was closely related to HNSCC immune cell infiltration and prognosis. However, there was no direct evidence that AVPR2 played a role in immune infiltration and thus affected prognosis. For confirmation of the biological function of AVPR2 in HNSCC, more in vivo/in vitro experiments are needed.

## Conclusion

In conclusion, we found that the AVPR2 gene is an independent prognostic factor for HNSCC, with patients who have high AVPR2 expression showing a better prognosis. In addition, AVPR2 may play a role in HNSCC immune modulation, and the regulation of tumour-infiltrating B cells by AVPR2 may be a key link.

## Data Availability

Several online tools were used to analyze data from the TCGA database, including cBioportal (http://www.cbioportal.org/), UALCAN (http://ualcan.path.uab.edu/analysis.html), GEPIA(http://gepia.cancer-pku.cn/detail.php), PrognoScan databases (http://dna00.bio.kyutech.ac.jp/PrognoScan/index.html), TISIDB (http://cis.hku.hk/TISIDB/search.php), TIMER (https://cistrome.shinyapps.io/timer/), LinkedOmics (http://www.linkedomics.org/login.php), STRING (https://cn.string-db.org/), CytoScape (https://cytoscape.org/download.html). In addition, dataset (GSE59102) from the Gene Expression Omnibus database (GEO, https://www.ncbi.nlm.nih.gov/geo/) were also analyzed.

## Abbreviations

HNSCC: Head and neck squamous cell carcinoma
TCGA: The Cancer Genome Atlas
GEO: Gene Expression Omnibus
AVPR2: Arginine vasopressin receptor 2
GPCR: G protein-coupled receptor
AVP: Arginine pressor
ICISs: Immune checkpoint inhibitors
Irae: Immune-related adverse events
GSEA: Gene set enrichment analysis
OS: Overall survival
DFS: Disease-free survival
RFS: Relapse-free survival
